# Femoral Fixation for Primary Total Hip Arthroplasty—An International Registry Perspective

**DOI:** 10.1016/j.artd.2025.101755

**Published:** 2025-06-23

**Authors:** Tony Gaidici, David G. Deckey, Thorsten M. Seyler, Michael P. Bolognesi, Mark J. Spangehl, Joshua S. Bingham

**Affiliations:** a University of Arizona College of Medicine, Phoenix, AZ, USA; b Department of Orthopaedic Surgery, Mayo Clinic, Phoenix, AZ, USA; c Department of Orthopaedic Surgery, Duke University Medical Center, Durham, NC, USA

**Keywords:** Total hip arthroplasty, THA, Femoral fixation, Cemented fixation, Uncemented fixation

## Abstract

**Background:**

The preferred method for femoral fixation (FF) during total hip arthroplasty (THA) has varied internationally. While prior studies explored trends within single countries or small international cohorts, there’s a paucity of studies comparing international trends of FF for primary THA patients. This study analyzed global trends in FF methods, survival by fixation type, and periprosthetic fractures as a reason for revision.

**Methods:**

Data were extracted from 2000 to the present from 16 national joint replacement registries. Data were collected on the number of cemented and uncemented primary THA cases each year. Revision and survival data were analyzed by fixation method. Aggregate data on periprosthetic fractures as a reason for revision and specific data on periprosthetic fractures by fixation method were included when available.

**Results:**

A global shift from cemented to uncemented FF was observed over the past two decades, except in the Netherlands. Even in countries favoring cementation, such as Sweden, uncemented techniques are gaining popularity. Survival rates for cemented and uncemented methods were comparable, but periprosthetic fracture rates varied regionally. Preferences reflected both clinical practice differences and patient needs.

**Conclusions:**

Most countries investigated show a preference for uncemented fixation. However, the popularity of cemented fixation in certain countries reflects its continued relevance, especially in populations with different clinical needs. Survival by fixation method was similar among countries, but periprosthetic fracture as reason for revision varied widely.

## Introduction

Globally, millions of patients undergo total hip arthroplasty (THA) each year, with the procedure's prevalence expected to increase significantly, especially within Organization for Economic Cooperation and Development countries [1]. In the United States between 2008 and 2018, the annual volume of THA increased by 26%, with projections estimating 850,000 procedures annually by 2030 [2,3]. Over the past 30 years in the United States, there has been an increasing trend toward uncemented femoral fixation (FF) in primary THA [4]. Uncemented FF was also favored over cemented FF when analyzing the registries of countries like England, Sweden, New Zealand, and Australia albeit to a relatively lesser degree, while Norway notably stood out as favoring the cemented technique [4].

Prior studies have investigated the prevalence of cemented vs uncemented FF for THA in individual countries. For instance, in the United States multiple studies indicate that uncemented FF is the most widely practiced technique in about 92%-98% of all THA cases [[4], [5], [6]]. A study by Sheridan et al. found that the UK, Australia, New Zealand, and Canada likewise predominantly favor uncemented FF [7], which is in concurrence with findings in the USA [4]. Another study found a strong preference for uncemented FF in South Korea [8]. Interestingly, in countries under the NARA registry, which includes Norway, Sweden, Denmark, and Finland, 67% of FF cases were cemented and only 21% were uncemented [9]. However, each of these studies investigated the registries of a single country or a very small number of countries. To date, no study has investigated and compared the international trends in FF for primary THA. The purposes of this study were to (1) analyze the trends in FF for primary THA internationally, (2) investigate trends in survival by fixation method, and (3) determine whether modes of failure varied by fixation method. We hypothesize that while there are regional variations in the adoption of cemented vs uncemented FF for THA, the global trend will demonstrate a shift toward uncemented fixation. Furthermore, we anticipate that, despite differences in fixation preference, survival rates will be comparable between the 2 techniques, but periprosthetic fracture rates will be higher in uncemented fixation.

## Material and methods

### Data collection

This study was exempt from institutional review board approval due to the use of publicly available and deidentified registry data. Data were extracted for the years 2000 to 2024 from the annual reports of arthroplasty data from the following 16 countries (abbreviated registry name in parentheses): Australia (AOANJR), Canada (CJRR), Catalonia/Spain (RACat), Denmark (DHR), Germany (EPRD), India (IJR), Italy (RIPO), Netherlands (LROI), New Zealand (NZJR), Norway (NAR), Romania (RAR), Slovakia (SAR), Sweden (SAR), Switzerland (SiRiS), United Kingdom (NJR), United States (AJRR) [[10], [11], [12], [13], [14], [15], [16], [17], [18], [19], [20], [21], [22], [23]]. These registries were chosen because of the availability of publicly accessible reports in English and the availability of procedural data on implant fixation techniques used for THA. Data were collected from publicly available annual registry reports, except for Canada’s, which had to be requested directly from the CJRR. While the Indian Joint Registry was not publicly available, fixation data from 2006 to 2019 were published in a review by Vaidya et al. [24]. Data collection encompassed the earliest reported year available from each registry after 2000 to the present. However, some registries commenced data acquisition after 2000 or had no records extending to this period. The following registries did not report fixation data in exact numerical figures but rather as chart data, requiring visual estimation to extract numerical fixation data: NZJR, AOANJR, SAR (Sweden), EPRD, and IJR. The AJRR only reported data on cemented FFs. For registries that also reported on hybrid fixations, hybrid and cemented FF were combined into a single figure (Table 1).

**Table 1:** Data availability of each country’s registry.

Country	Primary THA cementation data	Survival by fixation type	Survival of all fixations	All periprosthetic fracture	Periprosthetic fracture by fixation type
Australia	Yes	Yes	Yes	Yes	Yes
Canada	No	Yes	Yes	Yes	No
Denmark	Yes	No	No	Yes	Yes
Germany	Yes	Yes	Yes	Yes	No
India	Yes	No	No	No	No
Italy	Yes	Yes	Yes	Yes	Yes
Netherlands	Yes	No	Yes	Yes	No
New Zealand	Yes	Yes	Yes	Yes	No
Norway	Yes	No	No	Yes	No
Romania	Yes	No	No	Yes	No
Slovakia	Yes	No	No	Yes	No
Spain	Yes	Yes	No	No	No
Sweden	Yes	No	Yes	Yes	No
Switzerland	No	Yes	No	Yes	Yes
UK	Yes	No	No	Yes	No
USA	Yes	No	No	Yes	Yes

### Data analyses

Data were divided by source registry (Table 1) into cemented and uncemented FF in primary THA representing categorical, independent groups using Microsoft Excel (Microsoft, Redmond, Washington). Data visualizations were generated using Python's Matplotlib library (version 3.5) (Mathworks, Nattick, Massachusetts), which allowed for detailed customization of chart elements.

## Results

**Figure 1:**
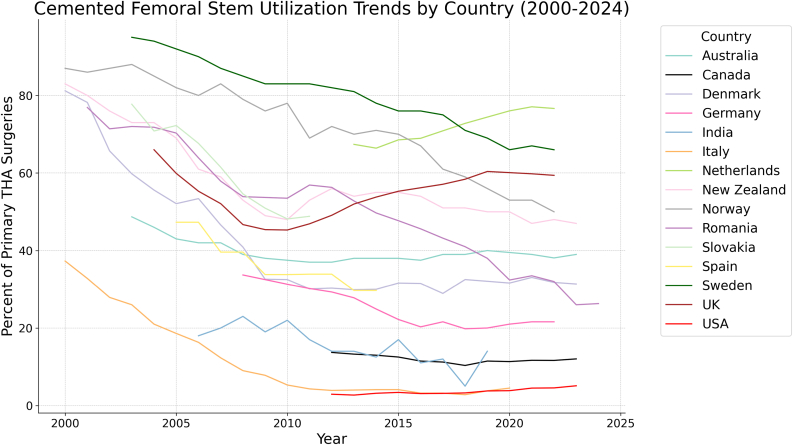
Cemented femoral stem utilization trends by country (2000-2024).

The rate of cemented FF varied considerably across countries from 2000 to 2024, with the United States reporting a low of 2.7% in 2013 and Sweden reporting an estimated high of 95% in 2003. Only 2 countries, the United States and the Netherlands, showed an increasing rate of cemented FF during this period, whereas India remained largely unchanged. The rest of the countries showed a decreased preference for cemented FF over the years. Among the countries showing a decline in cemented FF, the initial rates ranged from 95% in Sweden (2003) to 76.9% in Romania (2001). The overall decline across this group varied, with Romania experiencing the most significant drop of 50.9 percentage points (from 76.9% in 2001 to 26% in 2023), followed by Sweden with a 34.9 percentage point reduction (from 95% in 2003 to 60.1% in 2022). The rate of decline, calculated as an annual percentage decrease, ranged from 1.75% per year in Sweden to 2.32% per year in Romania. The greatest increase was noted in the Netherlands, rising from 67.4% to 76.7% from 2013 to 2022. The United Kingdom had the greatest relative rise in cementation from a low of 45.3% to 60.4% from 2010 to 2019 (due to its increasing preference for hybrid cemented fixations); however, by expanding the date range to encompass all its reported years from 2004 to 2022, it still showed an absolute decrease in cementation rate, from 66% to 59.4% (Table 2 and Fig. 1).

**Table 2:** Proportion of cemented FF for primary THA by year.

Year	Australia	Canada	Denmark	Germany	India	Italy	Netherlands	New Zealand	Norway	Romania	Slovakia	Spain	Sweden	UK	USA
2000			*81.2*			*37.3*		*83*	87						
2001			78.2			32.8		80	86	*76.9*					
2002			65.7			27.9		76	87	71.4					
2003	*48.7*		59.8			26		73	*88*	72	*77.7*		*95*		
2004	46		55.6			21		73	85	71.8	70.8		94	*66*	
2005	43		52.1			18.6		69	82	70.3	72.2	*47.3*	92	59.9	
2006	42		53.4		18	16.3		61	80	63.9	67.6	*47.3*	90	55.3	
2007	42		46.6		20	12.3		59	83	57.9	61.5	39.6	87	52.1	
2008	39		40.9	*33.7*	*23*	9		53	79	53.9	54.6	39.6	85	46.7	
2009	38		32.6	32.5	19	7.8		49	76	53.7	50.9	33.8	83	45.4	
2010	37.5		32.5	31.3	22	5.3		48	78	53.5	**48.1**	33.8	83	**45.3**	
2011	**37**		30.1	30.2	17	4.3		53	69	56.9	48.8	33.9	83	46.9	
2012	**37**	*13.7*	30.3	29.3	14	3.9		56	72	56.3		33.9	82	49.1	2.92
2013	38	13.26	**29.9**	27.8	14	4	**67.39**	54	70	52.8		**29.7**	81	52	**2.7**
2014	38	12.98	30	24.9	12.5	4.1	66.41	55	71	49.7		**29.7**	78	53.8	3.17
2015	38	12.51	31.6	22.2	17	4.1	68.55	55	70	47.7			76	55.3	3.39
2016	37.5	11.47	31.5	20.3	11	3.2	68.95	54	67	45.6			76	56.2	3.09
2017	39	11.22	28.93	21.6	12	3.2	70.88	51	61	43.2			75	57.1	3.14
2018	39	10.34	32.51	**19.8**	**5**	**2.8**	72.8	51	59	41			71	58.4	3.26
2019	40	11.47	32.07	20	14	3.8	74.42	50	56	38			69	60.4	3.77
2020	39.5	**11.34**	31.61	21		4.5	76.03	50	53	32.4			**66**	60.1	3.84
2021	39	11.67	33.06	21.6			*77.08*	**47**	53	33.5			67	59.8	4.5
2022	38.1	11.64		21.6			76.65	48	**50**	32				59.4	*4.55*
2023		12.03								2**6**					
2024										26.3					

Lowest values per country denoted by bold font, and highest values for each country denoted by italic font.

**Figure 2:**
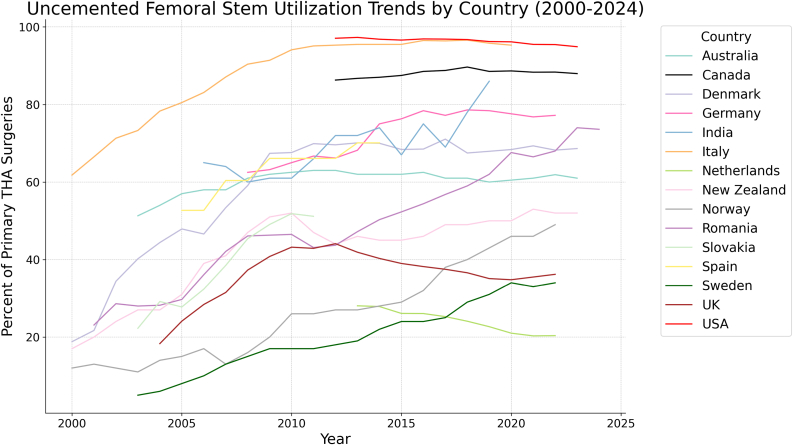
Uncemented femoral stem utilization trends by country (2000-2024).

The rate of uncemented fixations also varied considerably between countries in this study, with Sweden reporting an estimated low of 5% in 2003 and America reporting a high of 97.3% in 2013. Only the United States and the Netherlands had overall decreasing rates of uncemented fixation in this period, though notably the UK demonstrated a reduced preference for uncemented FF from 2010 to 2022 (43.2% to 36.2%) despite an absolute increase in uncemented FF use from 2004 to 2022 (18.3% to 36.2%). The greatest increase was noted in Romania, rising from 23.1% to 74% from 2001 to 2023. The greatest decrease was noted in the Netherlands, dropping from 28.1% to 20.4% from 2013 to 2022 (Table 3 and Fig. 2).

**Table 3:** Proportion of uncemented FF for primary THA by year.

Year	Australia	Canada	Denmark	Germany	India	Italy	Netherlands	New Zealand	Norway	Romania	Slovakia	Spain	Sweden	UK	USA
2000			**18.8**			**61.8**		**17**	12						
2001			21.7			66.5		20	13	**23.1**					
2002			34.4			71.3		24	12	28.6					
2003	**51.3**		40.2			73.3		27	**11**	28	**22.26**		**5**		
2004	54		44.4			78.3		27	14	28.2	29.16		6	**18.3**	
2005	57		47.9			80.5		31	15	29.7	27.76	**52.7**	8	24.1	
2006	58		46.6		65	83.1		39	17	36.1	32.36	**52.7**	10	28.4	
2007	58		53.4		64	87.1		41	13	42.1	38.50	60.4	13	31.5	
2008	61		59.1	**62.5**	**60**	90.4		47	16	46.1	45.39	60.4	15	37.3	
2009	62		67.4	63.2	61	91.4		51	20	46.3	49.07	66.1	17	40.8	
2010	62.5		67.6	65	61	94.1		52	26	46.5	*51.85*	66.1	17	43.2	
2011	*63*		69.9	66.7	66	95.1		47	26	43.1	51.17	66.1	17	42.9	
2012	*63*	**86.3**	69.6	66.2	72	95.3		44	27	43.7		66.1	18	*44.1*	97.08
2013	62	86.74	70.1	68.2	72	95.5	*28.06*	46	27	47.2		*70.1*	19	41.9	*97.3*
2014	62	87.02	70	75	74	95.5	27.87	45	28	50.3		*70.1*	22	40.3	96.83
2015	62	87.49	68.4	76.3	67	95.5	26.09	45	29	52.3			24	39	96.61
2016	62.5	88.53	68.5	78.4	75	96.5	26.06	46	32	54.4			24	38.2	96.91
2017	61	*88.78*	*71.07*	77.2	69	96.4	25.27	49	38	56.8			25	37.5	96.86
2018	61	89.66	67.49	*78.6*	78	*96.6*	24.08	49	40	59			29	36.6	96.74
2019	60	88.53	67.93	78.4	*86*	95.8	22.67	50	43	62			31	35.1	96.23
2020	60.5	88.66	68.38	77.6		95.3	21	50	46	67.6			*34*	34.8	96.16
2021	61	88.33	66.93	76.8			**20.3**	*53*	46	66.5			33	35.5	95.5
2022	61.9	88.36		77.2			20.36	52	*49*	68				36.2	**95.45**
2023		87.97								*74*					
2024										73.6					

Lowest values per country denoted by bold font, and highest values for each country denoted by italic font.

**Figure 3:**
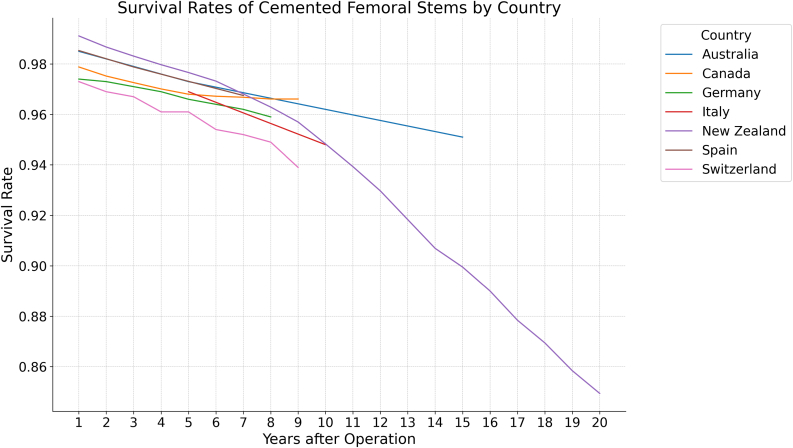
Survival rates of cemented femoral stems by country.

**Figure 4:**
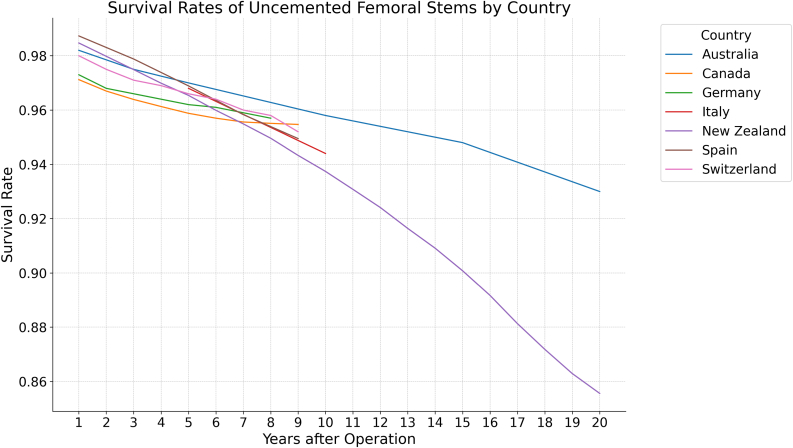
Survival rates of uncemented femoral stems by country.

**Figure 5:**
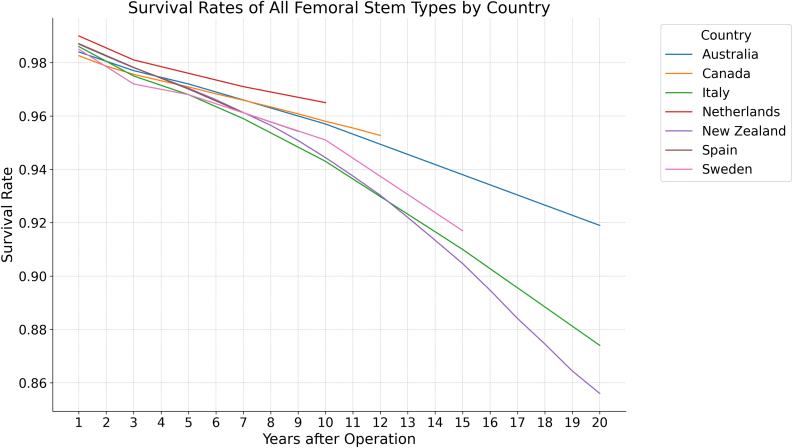
Survival rates of all femoral stem types by country.

Seven of the countries reviewed in this study reported on the survival of the hip prosthesis broken down by fixation type—New Zealand, Australia, Italy, Germany, Canada, Spain, and Switzerland. In the first 10 years postoperatively, survival by fixation type did not vary greatly between the countries studied. Only New Zealand and Australia reported beyond the 10-year mark, with New Zealand demonstrating a 5% lesser survival rate of cemented FF compared to Australia at the 15-year mark. At the 15 and 20-year marks, New Zealand reported a 4% and 8% lower survival rate of uncemented FF compared to Australia, respectively (Table 4, Figure 3, Figure 4, Figure 5).

**Table 4:** Survival of fixations. Switzerland only reported cemented and uncemented FF, while Sweden and the Netherlands only reported aggregate fixation survival.

Years after Operation	Australia			Canada			Germany			Italy			Netherlands	New Zealand			Spain			Sweden	Switzerland	
	Cem.	Uncem.	All	Cem.	Uncem.	All	Cem.	Uncem.	All	Cem.	Uncem.	All	All	Cem.	Uncem.	All	Cem.	Uncem.	All	All	Cem.	Uncem.
1	0.99	0.98	0.98	0.98	0.97	0.99	0.97	0.97	0.98			0.99	0.99	0.99	0.98	0.99	0.99	0.99	0.99	0.99	0.97	0.98
2				0.98	0.97		0.97	0.97	0.98					0.99	0.98	0.98					0.97	0.98
3	0.98	0.98	0.98	0.97	0.96	0.98	0.97	0.97	0.98			0.98	0.98	0.98	0.97	0.98	0.98	0.98	0.97	0.97	0.97	0.97
4				0.97	0.96		0.97	0.96	0.97					0.98	0.97	0.97					0.96	0.97
5	0.97	0.97	0.97	0.97	0.96	0.97	0.97	0.96	0.97	0.97	0.97	0.97	0.98	0.98	0.97	0.97	0.97	0.97	0.97	0.97	0.96	0.97
6				0.97	0.96		0.96	0.96	0.97					0.97	0.96	0.97					0.95	0.96
7				0.97	0.96	0.96	0.96	0.96	0.97			0.96	0.97	0.97	0.95	0.96	0.97	0.96			0.95	0.96
8				0.97	0.96		0.96	0.96	0.96					0.96	0.95	0.96					0.95	0.96
9				0.97	0.95	0.95			0.96					0.96	0.94	0.95		0.95			0.94	0.95
10	0.96	0.96	0.96						0.96	0.95	0.94	0.94	0.97	0.95	0.94	0.94			0.95	0.95		
11									0.96					0.94	0.93	0.94						
12									0.95					0.93	0.92	0.93						
13														0.92	0.92	0.92						
14														0.91	0.91	0.91						
15	0.95	0.95	0.94									0.91		0.90	0.90	0.90			0.92	0.92		
16														0.89	0.89	0.89						
17														0.88	0.88	0.88						
18														0.87	0.87	0.87						
19														0.86	0.86	0.86						
20		0.93	0.92									0.87		0.85	0.86	0.86						

Cem, cemented; Uncem, uncemented.

**Figure 6:**
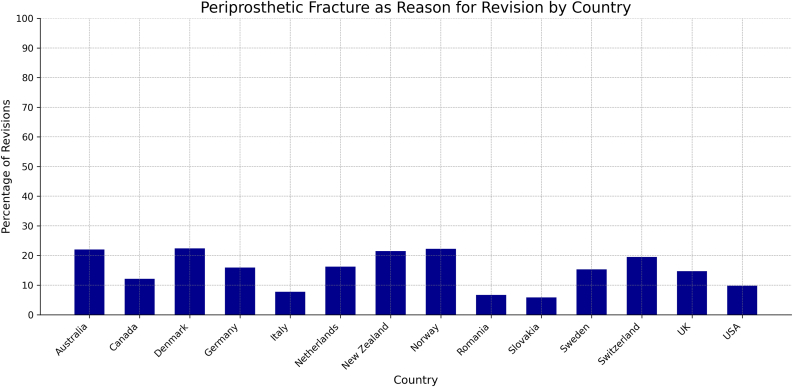
Periprosthetic fracture as a reason for revision by country.

**Figure 7:**
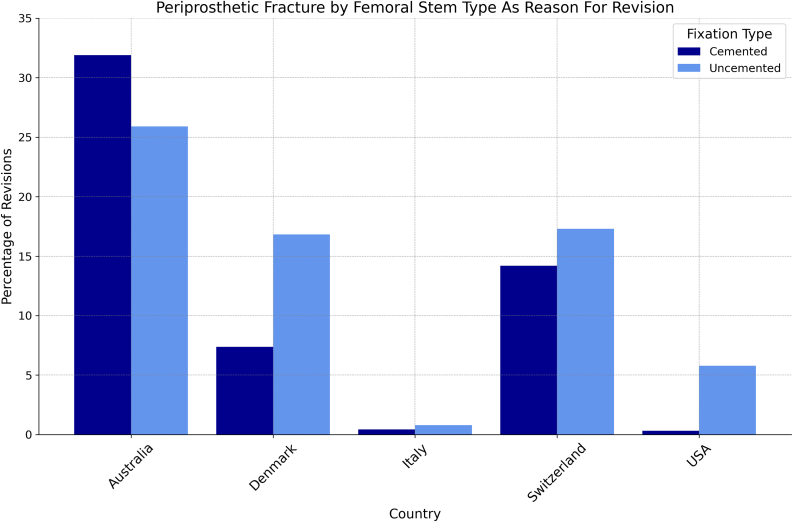
Periprosthetic fracture by femoral stem type as reason for revision.

Most of the countries investigated, except India and Spain, reported on periprosthetic fracture as reason for revision of primary THA. The percentage of revision surgeries due to periprosthetic fracture did not vary greatly between the countries investigated, with the largest gap being about 15.7% between Romania (6.7%) and Denmark (22.4%). However, when analyzed by fixation type, the gap widens within and between countries. Of note, all countries except for Australia reported a higher proportion of periprosthetic fractures in patients with uncemented fixations compared to those with cemented fixations. The largest gap was noted in Denmark (9.5%) (Table 5, Table 6, Figure 6, Figure 7).

**Table 5:** Proportion of revision THAs due to periprosthetic fracture.

	AUS	CAD	DNK	DEU	ITA	NLD	NZL	Nor	ROU	SVK	SWE	CHE	GBR	USA
PPFx as Reason for Revision	0.22	0.12	0.22	0.16	0.08	0.16	0.22	0.22	0.07	0.06	0.15	0.20	0.15	0.10

AUS, Australia; CAD, Canada; DNK, Denmark; DEU, Germany; ITA, Italy; NLD, The Netherlands; NZL, New Zealand; NOR, Norway; ROU, Romania; SVK, Slovakia; SWE, Sweden; CHE, Switzerland; GBR, United Kingdom; PPFx, periprosthetic fracture.

**Table 6:** Proportion of revision THA’s due to periprosthetic fracture, by fixation type.

Fixation type	Australia	Denmark	Italy	Switzerland	USA
Cemented	0.32	0.07	0.004	0.14	0.003
Uncemented	0.26	0.17	0.078	0.17	0.06

## Discussion

The data included in this study, spanning approximately 2 decades, highlight a noticeable shift from cemented to uncemented FF during THA internationally, with 1 notable exception, the Netherlands. Even in countries where cemented FF continues to be the most popular, such as in Sweden, the trend indicates an increasing popularity of uncemented fixation. This trend aligns with prior studies reporting on the increasing use of uncemented fixation. In the USA, multiple studies indicate that uncemented FF is the most widely practiced technique at about 92%-98% of all THA cases [[4], [5], [6]], which is reflected in the AJRR registry data.

Sweden and the Netherlands continue to favor cemented fixations. In Sweden, despite a gradual increase in the use of uncemented stems, cemented femoral stems remain dominant. This preference, especially in northern Europe and Scandinavia in particular, may be due to historical and long-established traditions of cemented techniques and their proven long-term outcomes in older populations, as noted by Murray in his investigation on the benefits of cemented fixation in elderly patients [25]. For comparison, Sweden reported older mean ages of 69 and 81.2 years for primary THA and THA due to fracture, respectively, while the USA reported younger ages of 65.6 and 71.8 for the same metrics, respectively. Therefore, it may be that Sweden favors cemented FF due to its relatively older patient population.

Older studies informally acknowledged a trend toward uncemented FF among hundreds of surgeons surveyed; however, they argued that choosing uncemented over cemented FF for THA, especially in the elderly, was paradoxical given the available data demonstrated superior outcomes with cemented FF in this patient population [[26], [27], [28], [29]]. This is now highly debated, with multiple recent studies showing superior overall outcomes, in particular with triple taper and collared stems among uncemented FF as compared to cemented FF, while others continue to purport the superiority of cemented FF in the elderly [[30], [31], [32]]. Notably, Moore et al. reported on over 62,000 patients and found a significantly lower incidence of infection, aseptic revision, venous thromboembolism, and aseptic loosening in uncemented FF compared to cemented FF [33].

Advocates for uncemented FF argue that using a cemented techniques can be technically challenging, increase surgery duration, and elevate the risk of aseptic loosening, especially in younger, more active patients [34]. In contrast, proponents of cemented FF emphasize its lower risk of reoperation, greater adaptability in patients with suboptimal bone quality, and reduced incidence of periprosthetic fractures and thigh pain, all contributing to improved long-term implant survival, particularly in elderly patients [5,25]. Notably, both the type of cemented femoral stem and type of acetabular component can significantly impact outcomes. In Australia, the top cemented stems in 2023 were the Exeter V40, Quadra-C, CPT, Taper Fit, and Short Exeter V40, while uncemented stems included the Accolade II and CORAIL. The survival rates varied by stem and acetabular component combination, with the Exeter V40-Exeter combination showing a 16.5% revision rate at 20 years, compared to 11.3% for the Exeter V40-Exeter Contemporary [10]. Additionally, differences in cementing techniques may impact survival. For example, the French paradox technique has been shown to have excellent outcomes, with a 17-year survival rate of 90.5% for all revisions [35]. In comparison, the Exeter stem, often considered the gold standard for older patients with osteoporotic bone, has been shown to have a 22-year survivorship of 74.9% for all revisions [36]. At present, there is no consensus among orthopaedic surgeons on the superiority of either technique, leading to clinical equipoise and a deference to individual surgeon preferences. In osteoporotic bone, however, there are clear and undisputable benefits to cemented femoral stem fixation over cementless femoral stem fixation regardless of cementing technique [37,38].

In this study, aggregate survival among cemented and uncemented fixations did not vary widely within the handful of countries that reported survival data. When analyzed by fixation type, there was a slightly lower survival rate among cemented fixations in Switzerland compared to the rest, but this difference is only by a few percentage points and is unlikely to suggest any meaningful variation in quality of care. There is still ongoing debate on whether cemented FF or uncemented FF confers higher long-term survivorship, with many papers suggesting uncemented FF may have superior survivability in younger adults whereas cemented FF may have superior survivability for older adults [5,8,39,40]. Surgeons may find the results of this study helpful in adjusting their own practice based ton demographic and institutional factors that influence fixation choices. For instance, surgeons practicing in regions with a higher proportion of older patients may continue to favor cemented fixation, as these techniques have demonstrated superior long-term outcomes in elderly populations with poor bone quality. However, as uncemented fixation continues to gain popularity, especially in younger, more active patients, surgeons may consider using these techniques in patients with better bone quality or those who are likely to benefit from enhanced implant longevity.

Periprosthetic fracture as the reason necessitating a revision surgery remained at less than 25% for all countries reporting these data. In the 5 registries that broke this down further by fixation type, all countries but Australia reported a higher rate of periprosthetic fracture as reason for revision in uncemented fixations as opposed to cemented fixations. Further research is warranted to investigate whether these differences are statistically significant. Regarding surgical approach, Australia reported lower revision rates for the anterior approach (2.5%) compared to the posterior (2.9%) and lateral approaches (3.1%) 5 years postoperative, while Switzerland showed lower revision rates for the lateral approach (2.0%) compared to anterior (2.5%) and posterior approaches (3.1%) at 2 years postoperative [10,21].

This study has multiple limitations. First, data extraction relied on publicly available registry reports, which have varying degrees of completeness and consistency across different countries. Additionally, not all national registries included captured all procedures in any given nation. For instance, while certain national registries capture nearly all arthroplasty performed in their country, registries such as the AJRR only capture a small proportion of the surgeries performed in the United States. Thus, although a trend can be seen in the AJRR, this may or may not be truly representative of the United States as a whole. Another limitation is the exclusion of patient-specific factors such as age, comorbidities, and bone quality, which could influence fixation choices and outcomes but are not consistently available in registry data across countries. Finally, because this study is retrospective in nature, causative conclusions regarding international fixation trends cannot be drawn. Future studies should incorporate a granular, patient-level demographic and surgical data analysis within and between countries to establish causation for the trends noted in this study and to assess the clinical outcomes in the different populations investigated. These studies should identify the factors driving fixation choices in each country such as healthcare system differences, surgeon training, and patient demographics, which would better help healthcare practitioners tailor their practices to the patient populations they serve.

## Conclusions

Most countries investigated show a preference for uncemented fixation. However, the persistent use of cemented fixation in certain countries reflects its continued relevance, especially in populations with different clinical needs. Survival outcomes were generally similar across countries regardless of fixation method, suggesting that both approaches can provide durable results when applied appropriately. Periprosthetic fractures as a reason for revision varied by country and fixation type, with uncemented fixations associated with higher fracture rates.

## Declaration of generative AI and AI-assisted technologies in the writing process

During the preparation of this work, the authors used ChatGPT-4o to assist with writing the Python code used for generating the figures as well as editing sentences for clarity and structure. After using this tool/service, the authors reviewed and edited the content as needed and take full responsibility for the content of the publication

## Conflicts of interest

David G. Deckey owns stock/stock options in Osgenic; has Medical/Orthopaedic publications editorial/governing board at Journal of Arthroplasty; and is a Board member/committee appointments for AAHKS and AAOS.

Thorsten M. Seyler receives royalties from Smith & Nephew, Restor3D, and Pattern Health; is a paid consultant for Smith & Nephew and Restor3D; owns stock or stock options at Restor3D, Extrel Therapeutics, and MiCarePath; receives research support from Zimmer Biomet; is on the Medical/Orthopaedic publications editorial/governing board at Wolters Kluwer; and is a Board member/committee appointments at Musculoskeletal Infection Society, American Association of Hip & Knee Surgeons.

Michael Bolognesi receives royalties from Zimmer Biomet, TJO, Smith & Nephew; is on the Speakers bureau/paid presentations for Ethicon; owns Stock or stock options at TJO; receives research support from Zimmer Biomet, Smith & Nephew, Stryker, Depuy Synthes; and is a member at AAHKS Board of Directors, EOA Board of Directors, SOA Board of Directors, and OREF Board of Directors.

Mark Spangehl's owns stock in Sonoran Biosciences, received research support from Stryker and Depuy, and is in the editorial board of *Journal of Arthroplasty*.

The other authors declare there are no conflicts of interest.

For full disclosure statements refer to https://doi.org/10.1016/j.artd.2025.101755.

## CRediT authorship contribution statement

**Tony Gaidici:** Writing – original draft, Investigation, Formal analysis, Data curation. **David G. Deckey:** Writing – review & editing, Project administration, Formal analysis, Conceptualization. **Thorsten M. Seyler:** Writing – review & editing. **Michael P. Bolognesi:** Writing – review & editing. **Mark J. Spangehl:** Writing – review & editing. **Joshua S. Bingham:** Writing – review & editing.

## Appendix A Supplementary data

Conflict of Interest Statement for Bingham

Conflict of Interest Statement for Bolognesi

Conflict of Interest Statement for Deckey

Conflict of Interest Statement for Gaidici

Conflict of Interest Statement for Seyler

Conflict of Interest Statement for Spangehl
