# Supplementary material for: Femoral Fixation for Primary Total Hip Arthroplasty—An International Registry Perspective
Source: Arthroplast Today. 2025 Jun 23;34:101755. doi: 10.1016/j.artd.2025.101755 (PMC12240149; doi:10.1016/j.artd.2025.101755)
Supplement: Conflict of Interest Statement for Seyler [file mmc5.docx]

# BLINDED CONFLICT OF INTEREST STATEMENT

***Arthroplasty Today***

(Adopted from the American Academy of Orthopaedic Surgeons disclosure statement)

The following form **must be filled out completely listing all author affiliations.**  **If no disclosure is required please write/type “none” at the end of each sentence.**

**Title: Femoral Fixation for Primary Total Hip Arthroplasty – An International Registry Perspective**

1. Royalties from a company or supplier (The following conflicts were disclosed)

**Smith & Nephew, Restor3D, Pattern Health**

2. Speakers bureau/paid presentations for a company or supplier (The following conflicts were disclosed)

**None**

3A. Paid employee for a company or supplier (The following conflicts were disclosed)

**None**

3B. Paid consultant for a company or supplier (The following conflicts were disclosed)

**Smith & Nephew, Restor3D**

3C. Unpaid consultants for a company or supplier (The following conflicts were disclosed)

**None**

4. Stock or stock options in a company or supplier (The following conflicts were disclosed)

**Restor3D, Extrel Therapeutics, MiCarePath**

5. Research support from a company or supplier as a Principal Investigator (The following conflicts were disclosed)

**ZimmerBiomet**

6. Other financial or material support from a company or supplier (The following conflicts were disclosed)

**None**

7. Royalties, financial or material support from publishers (The following conflicts were disclosed)

**None**

8. Medical/Orthopaedic publications editorial/governing board (The following conflicts were disclosed)

**Wolters Kluwer**

9. Board member/committee appointments for a society (The following conflicts were disclosed)

**Musculoskeletal Infection Society, American Association of Hip & Knee Surgeons**

**Each author must sign AND print or type his/her name, date and submit a separate form**

In addition, one BLINDED Conflict of Interest form (no author names used) should be submitted per manuscript with all author disclosures.

Thorsten M. Seyler, MD PhD 11/10/24


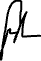

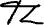


Author Name (Print or Type) Author Signature Date
